# Discrepancy-Guided Semantic Segmentation with Boundary Detail Enhancement for Traffic Scenes

**DOI:** 10.3390/s26092738

**Published:** 2026-04-28

**Authors:** Changshun Yu, Xiujian Yang, Shiquan Shen

**Affiliations:** 1 Faculty of Mechanical and Electrical Engineering, Kunming University of Science and Technology, Kunming 650500, China; y1713027396@163.com; 2 Faculty of Transportation Engineering, Kunming University of Science and Technology, Kunming 650500, China

**Keywords:** semantic segmentation, deep learning, boundary detail enhancement, discrepancy-aware fusion, traffic scene understanding

## Abstract

To address the challenges of missing fine-grained objects, blurred boundaries, and the suppression of shallow details by deep semantic features during cross-scale fusion in traffic scene semantic segmentation, this paper proposes a discrepancy-guided semantic segmentation method with boundary detail enhancement. First, to improve the semantic completeness of fine-grained regions, a Gated Collaborative Context Module (GCCM) is introduced between the encoder and decoder. By leveraging gating-guided channel selection and multi-scale contextual modeling, GCCM adaptively captures semantic dependencies across different scales. Second, to alleviate boundary ambiguity and detail loss, a Frequency–Edge Guided Enhancement Module (FEGE) is designed in the decoder. This module explicitly models low-frequency structural information and high-frequency edge components via frequency decomposition, and further enhances high-frequency details using the Scharr operator and lightweight convolution, thereby improving the structural representation of object contours and boundary regions. Furthermore, to mitigate the suppression of shallow details during cross-scale feature fusion, a Discrepancy-aware Pixel-Adaptive Gating Fusion module (D-PagFM) is proposed. By jointly modeling feature similarity and local discrepancy, the module adaptively regulates pixel-wise fusion, enhancing detail integration in structurally consistent regions while suppressing misleading fusion in inconsistent regions, thereby improving the robustness of feature fusion and boundary consistency. Experimental results on the Cityscapes and CamVid datasets demonstrate that the proposed method achieves mIoU scores of 80.08% and 82.97%, respectively. Moreover, it shows more significant improvements in boundary-sensitive fine-grained categories such as road boundaries, poles, and traffic signs, indicating its effectiveness and application potential for high-precision semantic segmentation in traffic scenes.

## 1. Introduction

Image semantic segmentation is an important research area in computer vision, aiming to assign a semantic category label to each pixel in an image. In traffic-scene perception, semantic segmentation serves as a key enabling technology for autonomous driving and intelligent transportation systems, providing essential semantic support for road structure understanding, traffic element recognition, as well as subsequent decision-making and path planning [1,2,3].

However, semantic segmentation in complex traffic scenes still faces three major challenges. First, fine-grained targets such as traffic signs, signal lights, and pole-like objects are prone to being missed during feature extraction due to their small scale and low pixel proportion. Second, repeated downsampling and feature smoothing tend to blur object boundary information, thereby weakening the model’s ability to represent structural details. Third, during cross-scale feature fusion, shallow detail information is easily suppressed by deep semantic features, which further affects segmentation accuracy. Therefore, how to effectively preserve fine-grained targets and boundary details while maintaining global semantic representation has become a key issue in traffic-scene semantic segmentation.

Although existing methods have achieved considerable progress in terms of accuracy, efficiency, and robustness for traffic-scene semantic segmentation, they still suffer from limitations in fine-grained target modeling, boundary detail preservation, and detail retention during cross-scale feature fusion, making it difficult to achieve an effective balance between global semantic representation and local structural details. To address these issues, this paper constructs a multi-module collaborative optimization framework spanning both the encoder and decoder stages from three aspects: fine-grained target semantic modeling, boundary detail enhancement, and cross-scale feature fusion regulation. Based on this, the main contributions of this paper are as follows:

1. A Gated Collaborative Context Module (GCCM) is proposed to adaptively regulate feature information flow by integrating a gating mechanism, multi-scale feature collaboration, and context completion strategies, thereby effectively alleviating the problems of incomplete semantic representation and insufficient boundary expression in fine-grained target regions.
2. A Frequency–Edge Guided Enhancement Module (FEGE) is proposed to combine frequency-domain decomposition with an edge-aware mechanism. Through explicit high-frequency guidance, the module achieves collaborative enhancement of structural preservation and boundary details, thereby improving the model’s responsiveness to high-frequency structural information and the accuracy of boundary representation.
3. A Discrepancy-aware Pixel-Adaptive Gating Fusion module (D-PagFM) is designed to adaptively regulate pixel-wise fusion regions by jointly modeling feature similarity and local discrepancy, thereby enhancing the robustness and prediction consistency of feature fusion in boundary regions, small-object areas, and occluded regions.

To better clarify the research background and technical positioning of this work, the following section reviews recent advances in traffic-scene semantic segmentation from three perspectives: improving segmentation accuracy, enhancing real-time performance, and strengthening cross-scene generalization ability.

## 2. Related Work

### 2.1. Research on Improving Segmentation Accuracy in Traffic Scenes

Such methods mainly improve segmentation accuracy through context modeling, multi-scale feature fusion, and boundary detail enhancement. In terms of context modeling, Wang et al. [4] proposed a context-aware segmentation network that incorporates Transformer attention mechanisms, enabling the model to effectively capture the relationship between small objects and global information, while further enhancing the representation of long-range small targets by introducing dilated convolutions. For multi-scale feature fusion, Xu and Zheng et al. [5] combined Atrous Spatial Pyramid Pooling (ASPP) with an attention mechanism to construct a multi-scale fusion framework, significantly improving the model’s perception of targets at different scales. Li et al. [6] proposed SANet, which introduces a selective context-encoding module and feature fusion strategy to effectively improve the recognition performance of tiny objects in street-scene segmentation. In addition, in terms of feature enhancement and loss optimization, Guo et al. In addition, Bai et al. [7] constructed a lightweight segmentation model based on a FasterNet backbone and a dilated residual pyramid structure, while Xie et al. [8] designed a lightweight Transformer-based semantic segmentation algorithm by integrating multi-scale deep convolution, both aiming to balance segmentation accuracy and model complexity in traffic scenes. Ref. [9] designed a boundary-aware loss function based on spatial adjacency relationships, which effectively improves the model’s ability to distinguish the boundaries of fine-grained targets. Gao et al. [10], from the perspective of multi-task modeling, proposed a cross-modal network framework integrating object detection and semantic segmentation, and enhanced the accuracy and robustness of small-object recognition in complex traffic scenes through joint optimization.

### 2.2. Research on Real-Time Semantic Segmentation for Traffic Scenes

In practical applications such as autonomous driving, real-time performance is a critical requirement for semantic segmentation systems. To this end, a large number of studies have focused on reducing computational complexity while maintaining segmentation accuracy. DDRNet [11] employs a dual-resolution architecture to jointly model high-resolution detail information and low-resolution semantic information, achieving favorable accuracy while preserving real-time performance. STDCNet [12] enhances feature representation through a short-term dense concatenation structure, enabling efficient feature extraction and fast inference. PIDNet [13], inspired by control theory, establishes a dynamic balance between accuracy and speed, thereby significantly improving real-time segmentation performance. In addition, lightweight segmentation models such as SCTNet [14] further improve segmentation accuracy while reducing computational cost by introducing efficient attention mechanisms and structural optimization strategies. Although these methods exhibit clear advantages in inference speed, they still show certain limitations in modeling fine-grained targets and boundary details.

### 2.3. Research on Cross-Scene Generalization for Traffic Scene Segmentation

With the continuous expansion of traffic-scene applications, semantic segmentation models are required not only to achieve high accuracy in standard road environments, but also to maintain stable performance across different cities, weather conditions, and imaging domains. Consequently, recent research has gradually extended from single-scene accuracy optimization to Domain Generalized Semantic Segmentation (DGSS), which aims to improve robustness across unseen scenarios [15,16,17]. DGSS seeks to train models on several source domains so that they can still achieve satisfactory segmentation performance on unseen target domains, thereby enhancing generalization capability and practical applicability. Existing studies mainly include technical routes such as the decomposition of domain-invariant and domain-specific features, meta-learning-based robust training, feature normalization and whitening, style transfer, and strong data augmentation, and have further evolved toward multi-granularity feature modeling, cross-domain feature alignment, and robust segmentation under adverse weather conditions. Although these methods have shown promising results in improving cross-scene adaptability, most of them still rely on relatively traditional backbone networks, and the potential of vision foundation models for domain generalized semantic segmentation remains underexplored. Overall, this line of research focuses more on improving robustness to domain shifts and scene adaptation, while paying relatively less attention to fine-grained targets, boundary details, and detail preservation during cross-scale feature fusion.

## 3. The Proposed Algorithm

**Figure 1:**
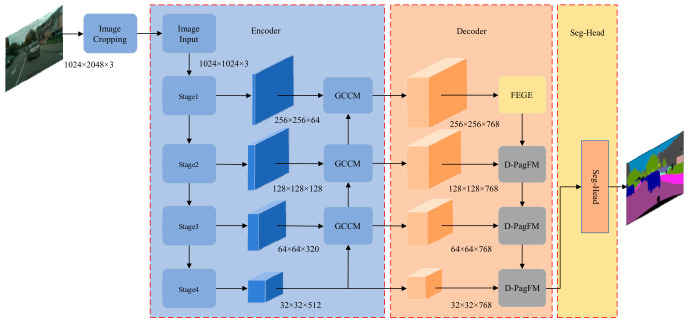
Overall Architecture of the Discrepancy-aware Boundary-enhanced Semantic Segmentation Method for Traffic Scenes.

To address the problems of missing fine-grained targets, blurred boundary details, and the suppression of shallow details by deep semantic features during cross-scale feature fusion in traffic-scene semantic segmentation, this paper proposes a discrepancy-aware boundary-enhanced semantic segmentation method for traffic scenes. The proposed method mainly consists of three key modules. First, a Gated Collaborative Context Module is introduced between the encoder and decoder to enhance the modeling of multi-scale semantic dependencies, thereby improving the semantic completeness of fine-grained target regions. Second, a Frequency–Edge Guided Enhancement Module is introduced in the shallow Stage 1 of the decoder to strengthen boundary structures and high-frequency detail information, thereby alleviating boundary ambiguity and detail loss. Finally, a Discrepancy-aware Pixel-Adaptive Gating Fusion module is designed in the decoder feature fusion stage to adaptively regulate features from different scales and suppress the interference of locally inconsistent features in the fusion process, thereby improving the robustness and boundary consistency of cross-scale feature fusion. The improved overall network architecture is illustrated in Figure 1. The proposed method adopts an encoder–decoder architecture, in which the encoder is responsible for extracting multi-scale features from the input image, while the decoder progressively restores spatial resolution and integrates semantic information with boundary details during the reconstruction process, ultimately generating pixel-level segmentation predictions.

### 3.1. Network Architecture Overview

As shown in the network architecture, the proposed method mainly consists of three parts: an encoder, a decoder, and a segmentation head. The encoder is composed of four stages, corresponding to four hierarchical Transformer Blocks, namely TB-1, TB-2, TB-3, and TB-4, which are used to progressively extract multi-scale feature representations from the input image. To enable effective aggregation of features from different encoder stages, a Gated Collaborative Context Module (GCCM) is introduced between the encoder and decoder. This module adopts a progressive aggregation strategy to collaboratively fuse multi-level features from deep to shallow stages, thereby promoting efficient cross-level information interaction and enhancing multi-scale feature representation.

In the decoder, a Frequency–Edge Guided Enhancement Module (FEGE) is first introduced at the shallow Stage 1 to strengthen the preservation of boundary structures and fine-grained details. Subsequently, the edge features extracted by FEGE at Stage 1, together with the semantic features output from different encoder stages, are jointly used to construct the fusion input and are fed into the Discrepancy-aware Pixel-Adaptive Gating Fusion module (D-PagFM). This fusion pathway preserves fine-grained boundary information while enabling the adaptive integration of multi-scale semantic features, thereby effectively improving both semantic representation and boundary-aware modeling of the feature maps. Such a fusion strategy not only enhances the representation of local boundary details, but also improves the robustness of multi-scale feature fusion through discrepancy-aware regulation, achieving collaborative optimization of detail preservation and semantic consistency.

#### 3.1.1. Encoder Pipeline

Given an input image of size H×W×3, an Overlapping Patch Embedding strategy is first employed to perform the initial representation of the image and map it into low-level visual tokens. These features are then fed into a Hierarchical Transformer Encoder for feature extraction. The encoder introduces an Efficient Self-Attention mechanism at each stage to reduce computational complexity while enhancing the modeling capability of long-range dependencies. The encoder constructs feature representations at four hierarchical levels, corresponding to 1/4, 1/8, 1/16, and 1/32 of the original input resolution, with output channel dimensions of 64, 128, 320, and 512, respectively, thereby forming a multi-scale, multi-semantic-level feature pyramid representation. The detailed workflow of the encoder is described as follows:

(1) $$x_{encoder}=\left\{\begin{array}{l} F_{stage\left(1\right)}\left(f^{\mathrm{TB}-1}\left(I\right)\right),I\in R^{H\times W\times C}\\ F_{stage\left(i\right)}=f^{\mathrm{TB}-i}\left(F_{stage\left(i-1\right)}\right),\left(i=2,3,4\right) \end{array}\right.$$

(2) $$f_{j}^{\prime }=f^{\mathrm{GCCM}}\left(F_{stage\left(j+1\right)},F_{stage\left(j\right)}\right),\left(j=3,2,1\right)$$

Here, *I* denotes the input image, *H × W* represents the spatial resolution of the feature map, and *C* is the number of feature channels; $x_{encoder}$ represents the output of the encoder; $f^{Transformer}\left(\cdot \right)$ denotes the feature map generated by the TB module; $F_{stage\left(i+1\right)}$ denotes the feature maps extracted from different stages of the encoder; $f^{GCCM}\left(\cdot \right)$ denotes the GCCM fusion; $f_{j}^{\prime }$ denotes the outputs of different encoder stages after GCCM fusion.

#### 3.1.2. Decoder with Edge Enhancement and Discrepancy-Aware Fusion

To enhance boundary representation in the shallow stages of the decoder and achieve adaptive fusion between high-resolution detail features and deep semantic features, this paper introduces a Frequency–Edge Guided Enhancement Module (FEGE) and a Discrepancy-aware Pixel-Adaptive Gating Fusion module (D-PagFM) into the decoder. Specifically, FEGE is used to explicitly compensate for boundary information and fine-grained structural details in the shallow stage, thereby strengthening the representation of high-frequency structures in the feature maps. D-PagFM is then employed in the cross-scale fusion stage to adaptively regulate features from different levels, suppressing the interference of locally inconsistent features and improving both the robustness and representational consistency of feature fusion. The detailed formulation of the decoder is given as follows:

(3) $$\left\{\begin{array}{l} f_{1}^{\prime \prime }=f^{\mathrm{FEGE}}\left(f_{1}^{\prime }\right)\\ f^{\text{D-PagFM}-2}=f^{\text{D-PagFM}}\left(f_{1}^{\prime \prime },f_{2}^{\prime }\right)\\ f^{\text{D-PagFM}-3}=f^{\text{D-PagFM}}\left(f^{\text{D-PagFM}-2},f_{3}^{\prime }\right)\\ f^{\text{D-PagFM}-4}=f^{\text{D-PagFM}}\left(f^{\text{D-PagFM}-3},f_{4}^{\prime }\right)\\ =x_{decoder} \end{array}\right.$$

Among them, f′ denotes the output of the encoder’s Stage 1 after passing through the FEGE module in the shallow Stage 1 of the decoder; $f^{\text{D-PagFM-i}},\left(i=2,3,4\right)$ and $x_{encoder}$ denote the feature output obtained through the D-PagFM module and the final output of the encoder, respectively; the remaining symbols have the same meanings as defined above.

#### 3.1.3. Construction of the Semantic Segmentation Head

To obtain refined segmentation results, the four output feature maps from the decoder (with a total of 768 channels) are first concatenated along the channel dimension. Subsequently, a fully connected layer is applied to reduce the feature dimensionality to 768. To mitigate overfitting, a regularization operation is further introduced. Finally, a 1 × 1 convolution layer is employed to project the features to the desired number of segmentation classes. The structure of the segmentation head can be formulated as follows:

(4) $$x_{seg-head}=UP\left(Conv_{1\times 1}\left(Drop\left(Linear\left(x_{decoder}\right)\right)\right)\right)$$

Among them, $x_{seg-head}$ denotes the output of the semantic output head; $UP\left(.\right)$ denotes the upsampling operation using the bilinear interpolation method; and $Linear\left(\cdot \right)$ denotes the fully connected layer.

### 3.2. Design of the Gated Collaborative Context Module

In traffic-scene semantic segmentation, fine-grained targets such as traffic signs, signal lights, and pole-like objects are prone to being overlooked during multi-scale feature fusion due to their small scale and weak feature responses, which often leads to incomplete semantic representation. Existing methods usually adopt a dual-branch architecture to separately model local details and global semantic information, and then realize information interaction through feature alignment and fusion. However, such methods generally lack an effective selective regulation mechanism during the fusion process, making it difficult to adaptively assign feature weights according to the semantic importance of different spatial locations, thereby limiting the representation capability for fine-grained targets.

To address this issue, this paper proposes a Gated Collaborative Context Module (GCCM), which introduces a gating mechanism into a conventional multi-scale fusion structure to enable the adaptive collaborative modeling of local detail features and global semantic information.

Specifically, GCCM introduces a gating factor along the channel dimension to characterize the dependency between local features and global semantics. By performing multi-scale context modeling on the input features and combining window-based self-attention with directional aggregation to extract global semantic information, the module generates collaborative contextual features that incorporate both local structural cues and global semantic representations. On this basis, a gating weight map is generated through a 1 × 1 convolution and a Sigmoid activation function. This weight map can adaptively regulate the information flow between local and global features according to the semantic response strength at different spatial locations.

In fine-grained target regions, the proposed gating mechanism enhances the selective attention to weak-response features while suppressing redundant background information, thereby reducing the risk that small targets are overlooked during feature fusion. In structural boundary regions, it further strengthens local detail representation and improves the model’s discriminative capability for complex structures. Finally, feature projection and fusion are performed through depthwise separable convolution and a channel MLP to obtain the output feature of the GCCM. The formulation of the proposed module is given as follows:

(5) $$\left\{\begin{array}{l} U=f_{loc}\left(F_{local}\right)+SA_{w}\left(F_{global}\right)+DA\left(SA_{w}\left(F_{global}\right)\right)\\ M=\sigma \left(f_{g}\left(U\right)\right)\\ F_{gccm}=F_{global}+\\ \mathrm{MLP}\left(F_{global}+f_{proj}\left(M⊙U+\left(1-M\right)⊙f_{loc}\left(F_{local}\right)\right)\right) \end{array}\right.$$

where $F_{global}$ denotes the global semantic branch from the backbone; $F_{local}$ denotes the local detail branch; $f_{loc}$ represents the local contextual features generated by 3 × 3 and 1 × 1 convolutions; $SA_{w}\left(\cdot \right)$ denotes the window-based self-attention; $DA\left(\cdot \right)$ represents the context aggregation along the row or column direction; U denotes the collaborative contextual features aggregated from local, global, and directional information; M denotes the gating weight map obtained via a 1 × 1 convolution followed by a Sigmoid activation; $f_{proj}\left(\cdot \right)$ represents the depthwise separable convolution projection; $\mathrm{MLP}\left(\cdot \right)$ denotes the channel MLP composed of pointwise convolutions; ⊙ denotes element-wise multiplication; and $F_{gccm}$ represents the final output feature of the GCCM.

**Figure 2:**
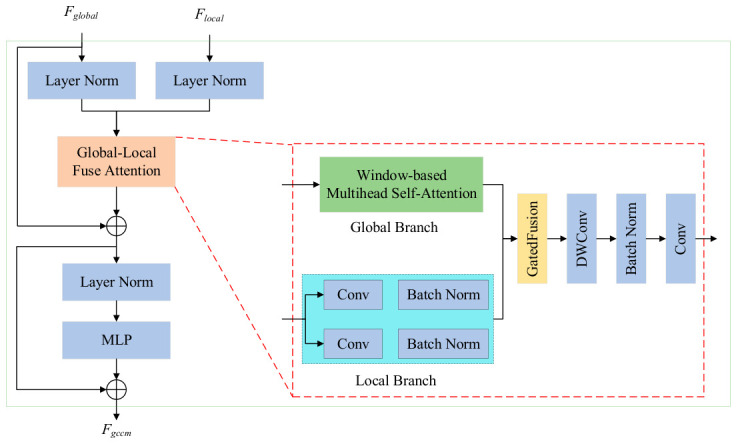
Gated Collaborative Context Module.

Equation (5) realizes a gated weighted fusion of local and global features, in which the gating factor is adaptively generated according to the importance of global features, thereby dynamically balancing local details and global context at different spatial locations. In fine-grained target regions, this mechanism enhances the selective attention to weak-response features and reduces the risk that small objects are overlooked during multi-scale fusion. In structural boundary regions, it further strengthens local detail representation and improves boundary discrimination capability. While maintaining a lightweight structure, the GCCM effectively enhances the model’s ability to represent fine-grained targets and complex structural regions. The structure of GCCM is illustrated in Figure 2.

### 3.3. Design of the Frequency–Edge Guided Enhancement Module

To effectively recover boundary weakening and fine-grained structural loss caused by multiple downsampling operations in the encoder during the decoding stage, a Frequency–Edge Guided Enhancement Module (FEGE) is proposed. The module simultaneously constructs low-frequency structural components and high-frequency edge components from the input features, enabling collaborative enhancement for structure preservation and edge compensation.

Specifically, FEGE decomposes the input features in the frequency domain. The low-frequency components are obtained via Gaussian depthwise convolution to preserve structural consistency in background regions such as roads and sky. In contrast, the high-frequency components are extracted using Scharr depthwise convolution to capture local intensity variations, and are further enhanced by a lightweight convolutional module for fine-grained detail refinement. To adapt to different semantic granularities across decoding stages, the intensity of the high-frequency components is adaptively adjusted according to the stage. As a result, the module focuses more on edge details in shallow layers, while emphasizing structural consistency in deeper layers.

In the local feature enhancement stage, FEGE takes the low-frequency structure as the backbone and explicitly guides local detail restoration using high-frequency edge information. This process compensates for the fine-grained geometric structures weakened during down sampling through local interaction and convolutional refinement.

Finally, to further improve representation capability while maintaining consistency with the optimization pathway of the backbone network, FEGE employs a lightweight 1 × 1 MLP for frequency-domain reconstruction. A residual connection is introduced to link the enhanced features with the input, ensuring stable feature enhancement without disrupting the original semantic distribution. In summary, the formulation of the proposed FEGE module can be expressed as follows:

(6) $$\left\{\begin{array}{l} X_{low}=G\left(X\right)\\ X_{edge}=\alpha C_{extra}\left(X_{x}^{2}+X_{y}^{2}\right)\\ X_{L}=\mathrm{BN}\left(X_{low}+\phi \left(X_{low}⊙X_{edge}\right)⊙w\right)\\ Y=X+\mathrm{BN}\left(DropPath\left(f_{\mathrm{MLP}}\left(X_{L}\right)\right)\right) \end{array}\right.$$

where $X\in R^{B\times C\times H\times W}$ denotes the input feature map; $G\left(\cdot \right)$ represents the Gaussian depthwise convolution with kernel size of 5 and standard deviation σ = 1.0; and $X_{x},X_{y}$ correspond to the depthwise convolution outputs of the Scharr−X/Y low-frequency and high-frequency components, respectively; $C_{extra}\left(\cdot \right)$ denotes the lightweight convolutional enhancement module; $\alpha \in \left(0,1\right]$ is the high-frequency weighting coefficient that is adaptively adjusted according to the decoding stage; ⊙ denotes element-wise interaction; $\phi \left(\cdot \right)$ represents the 3 × 3 convolutional refinement operator; $w\in R^{B\times C\times 1\times 1}$ denotes the channel attention weights generated by global average pooling (GAP) followed by a 1D convolution; $f_{\mathrm{MLP}}\left(\cdot \right)$ represents the lightweight MLP composed of two 1 × 1 convolution layers; and DropPath denotes the stochastic depth regularization mechanism.

**Figure 3:**
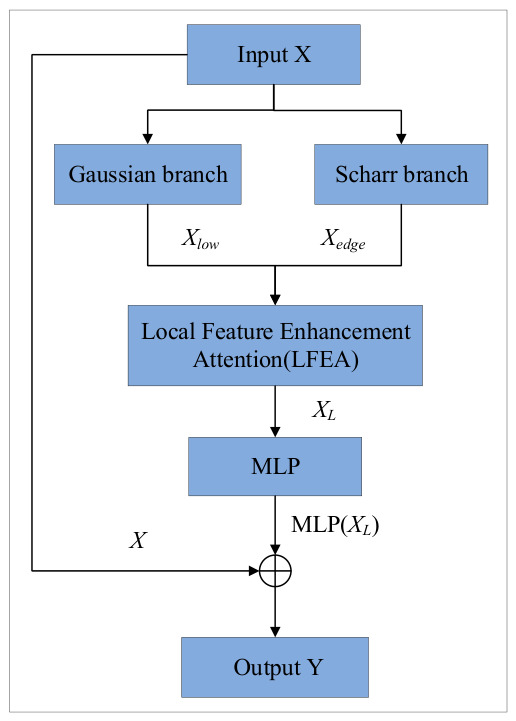
Frequency–Edge Guided Enhancement Module.

The structural diagram of the FEGE module is shown in Figure 3.

### 3.4. Design of the Discrepancy-Aware Pixel-Adaptive Gating Fusion Module

Conventional feature fusion methods in multi-scale semantic segmentation often lack a fine-grained fusion regulation mechanism, causing shallow detail features to be suppressed when fused with deep semantic features, thereby making it difficult for the model to balance spatial detail representation and semantic consistency [18,19,20,21]. To alleviate this issue, PagFM introduces a pixel-level gating mechanism that adaptively adjusts the fusion ratio between high- and low-resolution features by calculating local similarity between cross-scale features, thereby improving feature alignment errors to some extent [13,22]. However, the fusion weights in PagFM are mainly constructed based on feature similarity, lacking explicit modeling of local inconsistency and unreliable regions. In target boundary areas, occluded regions, and fine-grained target regions, where feature representations are unstable or semantic conflicts are more likely to occur, relying solely on similarity may lead to the misleading introduction of shallow details, resulting in detail suppression or erroneous fusion.

To address the above issue, this paper proposes a Discrepancy-aware Pixel-Adaptive Gating Fusion module (D-PagFM) based on PagFM. Unlike the original PagFM, which relies only on feature similarity for fusion, the proposed method introduces feature discrepancy modeling to characterize local feature inconsistency, thereby explicitly suppressing unreliable regions and improving the robustness of feature fusion.

Specifically, D-PagFM preserves the feature similarity computed by point-wise multiplication as a consistency indicator, while introducing feature differencing to characterize local inconsistency and using it as an indicator of unreliable regions. By introducing a learnable fusion weighting factor, the module achieves an adaptive balance between similarity and discrepancy to construct a spatial gating map, thereby enabling fine-grained regulation of pixel-wise fusion regions: in regions with high similarity, the injection of shallow detail features is enhanced to supplement structural information; in regions with high discrepancy (such as boundary-conflict regions, small-object regions, and occluded regions), excessive injection of shallow details is suppressed, thereby avoiding erroneous fusion and semantic conflicts.

This mechanism can effectively preserve shallow detail information in fine-grained target regions and alleviate its suppression by deep semantic features, while reducing the interference of inconsistent features in boundary regions, thereby improving the robustness and representational stability of feature fusion in structurally complex regions. In summary, while maintaining the lightweight advantage of the original method, D-PagFM introduces a discrepancy-aware fusion regulation mechanism to effectively suppress unreliable features and adaptively enhance detail information, thereby significantly improving feature fusion accuracy in boundary regions, small-object regions, and complex texture regions. The formulation of the proposed module is given as follows:

(7) $$\left\{\begin{array}{l} X_{k}=f_{x}\left(X\right)\\ Y_{q}=f_{y}\left(U\left(Y\right)\right)\\ S=W_{s}\left(X_{k}⊙Y_{q}\right)\\ D=W_{d}\left(\left|X_{k}-Y_{q}\right|\right)\\ G=\sigma \left(\alpha S+\left(1-\alpha \right)\left(1-\sigma \left(D\right)\right)\right)\\ F_{out}=\left(1-G\right)⊙X+G⊙Y \end{array}\right.$$

In Equation (7), $X,Y\in R^{B\times C\times H\times W}$ denote the deep semantic feature and the shallow detail feature, respectively; $U\left(\cdot \right)$ denotes the deep feature aligned to scale s through bilinear interpolation; $f_{x}\left(\cdot \right),f_{y}\left(\cdot \right)$ denotes the channel reduction operator composed of a 1 × 1 convolution and Batch Normalization (BN), which maps the input into an intermediate channel space $X_{k},Y_{q}\in R^{B\times C_{m}\times H\times W}$; S denotes the similarity response map constructed from point-wise multiplication $X_{k}⊙Y_{q}$ and further transformed by a 1 × 1 convolution, which is used to characterize the consistency of cross-scale features; D denotes the discrepancy response map generated from feature differencing $\left|X_{k}-Y_{q}\right|$ and further transformed by a 1 × 1 convolution, which is used to describe local representational inconsistency; $W_{s}\left(\cdot \right),W_{d}\left(\cdot \right)$ denote the mapping functions applied to the similarity term Sim_Conv and the discrepancy term Diff_Conv, respectively; $\sigma \left(\cdot \right)$ denotes the Sigmoid activation function; $\alpha \in \left[0,1\right]$ is a learnable scalar, and 1−α is its complementary weight, which is used to adaptively balance the contributions of the similarity response and discrepancy response in the generation of the gating map; $1-\sigma \left(D\right)$ denotes the normalized discrepancy response map, where larger discrepancy values indicate lower local feature consistency; $G\in {\left[0,1\right]}^{B\times C\times H\times W}$ denotes the final discrepancy-aware gating map; and $F_{out}$ denotes the fused output feature of the D-PagFM module.

**Figure 4:**
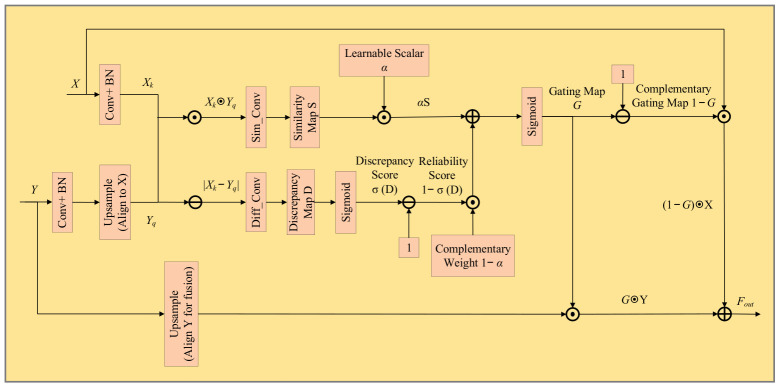
Discrepancy-aware Pixel-Adaptive Gating Fusion Module.

In Equation (7), when the gating weight G approaches 0, the fusion result tends to preserve deeper semantic features; when G approaches 1, the shallower detail feature $U\left(Y\right)$ is incorporated. In regions with high local feature consistency, the similarity response dominates the generation of the gating map, which facilitates the effective supplementation of shallow details. In contrast, in regions with boundary conflicts, occlusion, or locally inconsistent representations, the discrepancy response suppresses the gating weight, thereby reducing the excessive injection of unreliable detail information and avoiding erroneous fusion and semantic conflicts. The structure of the proposed D-PagFM module is illustrated in Figure 4, where α and 1−α denote a learnable scalar and its complementary weight, respectively.

## 4. Experimental Results and Analysis

To comprehensively evaluate the effectiveness of the proposed method, this section first introduces the datasets and evaluation metrics used in the experiments. Subsequently, the hardware environment and training configurations are described in detail. Systematic ablation studies are conducted on the Cityscapes dataset to analyze the contribution of each module to the overall performance. Furthermore, to validate the overall performance of the proposed approach, comparative experiments are carried out against state-of-the-art methods on both the Cityscapes and CamVid datasets. In addition, the per-class mIoU results of the proposed method before and after improvement are compared on both datasets. Finally, qualitative visualization results are provided to further demonstrate the superiority and practical value of the proposed method in semantic segmentation tasks.

### 4.1. Datasets and Evaluation Metrics

The Cityscapes dataset [23] is collected from street driving scenes across 50 different cities, with an original image resolution of 2048 × 1024 pixels. It contains 19 common traffic scene classes and is widely used for performance evaluation in road scene semantic segmentation tasks. The dataset includes approximately 20,000 coarsely annotated images and 5000 finely annotated pixel-level images. The finely annotated subset is further divided into training, validation, and test sets, consisting of 2975, 500, and 1525 images, respectively. In this work, the model is trained and evaluated based on the finely annotated data, and the original images are randomly cropped to 1024 × 1024 pixels as network inputs.

The CamVid dataset [24] is specifically designed for semantic segmentation in road scenes and provides pixel-level semantic annotations. Compared with Cityscapes, it is relatively small, containing a total of 701 annotated images, which are split into training, test, and validation sets with 367, 233, and 101 images, respectively. The image resolution is 960 × 720 pixels. Although the dataset defines 32 semantic classes, only 11 classes are selected for evaluation in this study.

To comprehensively evaluate the model’s semantic segmentation performance across different traffic scenes, four metrics are selected as evaluation criteria: Pixel Accuracy (PA), Mean Pixel Accuracy (MPA), Mean Intersection over Union (mIoU), and the number of model parameters (Parameters).

(1) Pixel Accuracy (PA) and Mean Pixel Accuracy (MPA)

Pixel Accuracy (PA) measures the overall classification accuracy of the model across all pixels, defined as the ratio of correctly classified pixels to the total number of pixels. Mean Pixel Accuracy (MPA) evaluates the average classification accuracy across all categories, providing a more comprehensive reflection of the model’s overall performance, especially on datasets with imbalanced class distributions. The calculation formulas are as follows:

(8) $$\left\{\begin{array}{l} PA=\frac{\sum_{i=1}^{K}n_{ii}}{\sum_{i=1}^{K}t_{i}}\\ MPA=\frac{1}{K}\sum_{i=1}^{K}\frac{n_{ii}}{t_{i}} \end{array}\right.$$

Among them, $n_{ii}$ denotes the number of pixels correctly classified as class i; $t_{i}$ represents the total number of pixels belonging to class i in the ground truth annotations; and *K* indicates the total number of classes.

(2) Mean Intersection over Union (mIoU)

Mean Intersection over Union (mIoU) is one of the most commonly used performance metrics in semantic segmentation. It measures the overlap between the predicted results and the ground truth for each category. It is defined as the average ratio of the intersection to the union of the predicted and ground truth regions across all categories. The calculation formula is as follows:

(9) $$mIoU=\frac{1}{K}\sum_{i=1}^{K}\frac{n_{ii}}{t_{i}+\sum_{j=1}^{K}n_{ji}-n_{ii}}$$

Among them, $n_{ii}$ denotes the number of pixels correctly classified as class *i*; $n_{ji}$ represents the number of pixels whose true class is *j* but are predicted as class *i*; $t_{i}$ indicates the total number of pixels belonging to class i in the ground truth; and the remaining symbols have the same meanings as above.

The above metrics enable a systematic analysis of the experimental results from both accuracy and model complexity perspectives.

### 4.2. Experimental Environment and Parameter Settings

The experiments in this study were conducted on an Ubuntu 20.04 64-bit operating system, using an Intel i7-13700KF CPU and an NVIDIA GeForce RTX 4090 GPU. The deep learning framework was built on PyTorch 1.12.0 and CUDA 11.3. To improve convergence speed and training stability, a SegFormer-B2 model pretrained on the ImageNet dataset [25] was used for initialization. During training, the total number of iterations was set to 160,000. The AdamW optimizer was adopted with an initial learning rate of $6\times 10^{-5}$, $\beta_{1}=0.9$, $\beta_{2}=0.999$, and a weight decay coefficient of 0.01. In addition, a poly learning rate decay strategy with 3000 warmup iterations was employed. The batch size was set to 1. In addition to random cropping, data augmentation strategies included random flipping, random rotation, and photometric distortion. Regarding the loss function, the main decode head employed a weighted combination of Cross-Entropy loss and Dice loss, where the weight of the Cross-Entropy loss was set to 0.4 and that of the Dice loss was set to 0.6. These settings were jointly adopted to improve the model’s learning ability for class imbalance, small targets, and boundary regions.

### 4.3. Ablation Experiments

To comprehensively validate the effectiveness of each functional module and its internal mechanism in the proposed model, two types of ablation experiments were conducted on the Cityscapes dataset: (1) inter-module ablation experiments, which were designed to evaluate the independent contribution of each functional module (GCCM, FEGE, and D-PagFM) to the overall performance; and (2) internal ablation experiments on the D-PagFM module, which were further conducted to analyze the role of its discrepancy-aware fusion mechanism. Through this two-level experimental design, the collaborative contributions of both the overall model architecture and the local fusion mechanism to performance improvement can be systematically validated. The results of the module ablation experiments on the Cityscapes dataset are shown in Table 1.

#### 4.3.1. Inter-Module Ablation Study

As shown in Table 1, taking SegFormer-B2 as the baseline model, the introduction of the Gated Collaborative Context Module (GCCM) between the encoder and decoder enables effective integration of multi-level features from deep to shallow layers through a top-down progressive fusion strategy. This design significantly enhances the model’s global semantic representation capability. With only an additional 3.76 M parameters, the overall pixel accuracy (PA) increases from 96.06% to 96.23%, the mean pixel accuracy (mPA) improves from 86.15% to 87.15%, and the mean Intersection over Union (mIoU) rises from 77.91% to 79.00%. These results demonstrate that the proposed GCCM achieves a favorable balance between performance improvement and computational cost. Considering that fine-grained structural information is often suppressed by high-level semantic features during cross-scale decoding, a Frequency–Edge Guided Enhancement Module (FEGE) is further introduced in the decoder branch to explicitly strengthen boundary responses and local structural representations. As shown in Table 1, when FEGE is applied independently without other modules, the number of parameters increases from 27.36 M to 35.21 M, and the mIoU improves from 77.91% to 78.96%, while PA and mPA increase to 96.22% and 86.43%, respectively. These results indicate that FEGE can consistently improve segmentation performance related to fine-grained structures, although its performance gains are accompanied by a certain increase in model complexity, mainly due to the additional enhancement branch and feature transformation operations.

During cross-layer feature fusion in the decoder, a Discrepancy-aware Pixel-Adaptive Gating Fusion module (D-PagFM) is introduced to adaptively select and weight shallow detail features and deep semantic features at the pixel level, thereby reducing the interference of inconsistent features in boundary-blurred regions, occluded areas, and semantic transition regions on the fusion results. When D-PagFM is introduced alone, the model parameters increase only to 29.14 M, while the mIoU rises to 79.50% and the mPA reaches 87.18%. This result indicates that, compared with FEGE, D-PagFM brings more significant fusion gains with a smaller increase in parameters, demonstrating a more positive effect in alleviating the accumulation of cross-scale alignment errors and improving feature fusion quality in object boundary regions and small-target areas.

Further combination ablation results demonstrate that the proposed modules exhibit complementary effects in improving segmentation performance. When FEGE and D-PagFM are introduced simultaneously, the model contains 37.00 M parameters, and the mIoU increases to 79.68%, achieving a 1.77% improvement over the baseline. This gain is significantly higher than that obtained by introducing each module individually. These results indicate that structural detail enhancement and pixel-level gated fusion can form effective synergy during cross-scale decoding, enabling both modules to better complement feature information. In the combination experiments involving GCCM, D-PagFM and FEGE are respectively integrated with GCCM. The results show that the model combining GCCM and D-PagFM contains 32.91 M parameters and achieves an mIoU of 79.35%, while the combination of GCCM and FEGE results in 38.97 M parameters and an mIoU of 79.42%. Although these two configurations differ in parameter scale and feature enhancement pathways, both effectively improve segmentation performance. This demonstrates that global context modeling, when combined with either local structural enhancement or pixel-level gated fusion, contributes positively to feature representation. When all three modules (GCCM, FEGE, and D-PagFM) are jointly incorporated, the number of parameters increases to 40.76 M, and the mIoU further improves to 80.08% (a gain of 2.17% over the baseline), with mPA reaching 87.95%. These results further validate the effectiveness of the multi-module collaborative design in complex urban scenarios. The proposed configuration enhances the representation of fine-grained structures and small-object regions while maintaining global semantic consistency, thereby significantly improving overall segmentation performance.

#### 4.3.2. Intra-Module Ablation Study of D-PagFM

After completing the inter-module ablation experiments and verifying the overall effectiveness of the improved fusion module, this paper further conducts internal ablation experiments on the D-PagFM module to analyze the specific roles and complementary relationship of the similarity branch and the discrepancy branch in pixel-wise gating generation, and to validate the effectiveness of the learnable weight α in adaptively balancing the two types of responses and improving fusion stability. Specifically, the complete D-PagFM is compared with the original PagFM, as well as with Sim-PagFM, which retains only the similarity branch, and Conf-PagFM, which retains only the discrepancy branch (where discrepancy/inconsistency is used as a proxy for fusion reliability), so as to quantitatively evaluate the influence of different gating compositions on feature fusion performance. The corresponding experimental results are summarized in Table 2 and Table 3, respectively.

As shown in Table 2, the baseline model achieves an mIoU of 77.91% on the Cityscapes dataset. After introducing the pixel-adaptive gating fusion mechanism (PagFM), the mIoU increases to 79.35%, indicating that the pixel-wise gating strategy can, to some extent, alleviate the problem that shallow details are overwhelmed by deep semantic features during multi-scale fusion, thereby improving the segmentation quality of object boundaries and small-target regions.

On this basis, when PagFM is further replaced with the proposed D-PagFM, the model mIoU increases to 79.50%, while the mPA improves from 87.05% to 87.18%. Although the number of parameters increases slightly, the overall performance becomes more stable. This indicates that, by introducing discrepancy modeling on top of similarity modeling and suppressing potential conflict regions through the gating mechanism, the proposed method can effectively reduce misleading fusion in semantic transition regions, boundary-conflict areas, and occluded regions, thereby improving the robustness and prediction consistency of pixel-wise feature fusion.

To further verify the independent roles of the similarity branch and the discrepancy branch in gating generation, and to analyze the advantage of joint modeling over single-branch modeling, finer-grained ablation experiments were designed for the internal structure of D-PagFM. Specifically, the performances of Sim-PagFM, which retains only the similarity response, and Conf-PagFM, which retains only the discrepancy response, were investigated separately. The corresponding results are summarized in Table 3.

As shown in Table 3, under the condition of identical parameter size, different gating compositions exhibit certain differences in segmentation accuracy and stability. When only a single branch is adopted, Sim-PagFM and Conf-PagFM achieve mIoU values of 79.27% and 79.13%, and mPA values of 86.86% and 86.52%, respectively, both of which are inferior to those of the jointly modeled Joint-PagFM. In contrast, by simultaneously introducing similarity response and discrepancy response, Joint-PagFM improves the mIoU to 79.41% and the mPA to 87.04%, indicating that the two types of responses exhibit good complementarity and can more effectively alleviate information mismatch and semantic conflicts during cross-scale fusion.

On this basis, the complete D-PagFM further introduces a learnable weight α to adaptively balance the similarity response and the discrepancy response, leading to a further improvement of mIoU to 79.50% and mPA to 87.18%, with more stable overall performance. These results indicate that a single-branch modeling strategy relying solely on either the similarity branch or the discrepancy branch is insufficient to consistently achieve superior pixel-wise fusion performance in complex traffic scenes. In contrast, the joint modeling of similarity and discrepancy, together with the adaptive balancing mechanism, can more fully exploit their complementary advantages, thereby improving the robustness and prediction consistency of cross-scale feature fusion.

### 4.4. Quantitative and Qualitative Analysis of Algorithm Performance

To validate the effectiveness of the proposed method, this paper compares the improved algorithm with several representative methods, including DeepLab-V3+ [26], BASeg [27], Mask2Former-R50 [28], STDC2-Seg100 [12], SegNeXt [29], SegFormer-B2 [30], and SegFormer-B3 [30], under a unified input resolution setting. To ensure the comparison is as objective as possible, a consistent input resolution is adopted, and both quantitative results and qualitative visualizations of different methods are comprehensively analyzed. For some reference results, the corresponding sources are specified in the table notes.

#### 4.4.1. Experimental Results on the Cityscapes Dataset

Under a unified input setting, quantitative comparisons between the proposed method and several representative approaches were conducted, and the results are reported in Table 4. As shown in Table 4, the proposed method achieves a PA of 96.37%, an mPA of 87.95%, and an mIoU of 80.08% on the Cityscapes dataset. Compared with methods such as DeepLab-V3+, STDC2-Seg100, and PIDNet-Small, the proposed method shows competitive segmentation performance, indicating its effectiveness in traffic-scene semantic segmentation. In particular, compared with the direct baseline SegFormer-B2, the proposed method improves the mIoU from 77.91% to 80.08%, yielding a gain of 2.17 percentage points. This demonstrates that the designed context modeling, boundary enhancement, and discrepancy-aware fusion mechanisms can effectively improve the segmentation quality of fine-grained targets and boundary regions.

It should be noted that some recent representative high-performance methods and larger variants within the same backbone family still achieve higher overall mIoU than the proposed method. For example, BASeg, SegFormer-B3, SegNeXt-S, and Mask2Former-Swin-B all report higher mIoU values in Table 4. Among them, SegFormer-B3, as a larger-scale model within the same SegFormer family as the baseline used in this work, has a stronger backbone capacity, while methods such as SegNeXt-S and Mask2Former-Swin-B rely on stronger backbones or more complex architectural designs. In contrast, the present work mainly focuses on targeted improvements within a relatively compact SegFormer-B2 encoder–decoder framework, aiming to address the problems of missing fine-grained targets, blurred boundary details, and the suppression of shallow details by deep semantic features during cross-scale fusion. Therefore, the value of the proposed method lies more in its targeted optimization for specific problems and its stable gains over the direct baseline, rather than in outperforming all recent high-performance methods as a whole.

Meanwhile, to further evaluate the performance differences across categories—particularly the capability of modeling thin structures such as poles, traffic signs, and road boundaries—a per-class mIoU comparison is conducted between the proposed method and the baseline SegFormer-B2 on the Cityscapes dataset. The corresponding results are presented in Table 5.

To further verify whether the performance improvements are primarily concentrated on thin and elongated objects rather than merely reflecting overall gains in large-area categories, a statistical analysis of per-class mIoU changes on the Cityscapes dataset is conducted. It can be observed that fine-grained categories such as poles and traffic signs generally achieve larger improvements under the proposed method, with an average mIoU increase of approximately 3%. In contrast, large-area categories such as road, buildings, and vegetation exhibit relatively smaller gains, typically within 1%. These results indicate that the performance improvements of the proposed method are mainly concentrated on thin structures and boundary-sensitive categories, rather than relying on overall optimization of large homogeneous regions. Instead, the method focuses on enhancing the representation and segmentation capability for fine-grained objects.

**Figure 5:**
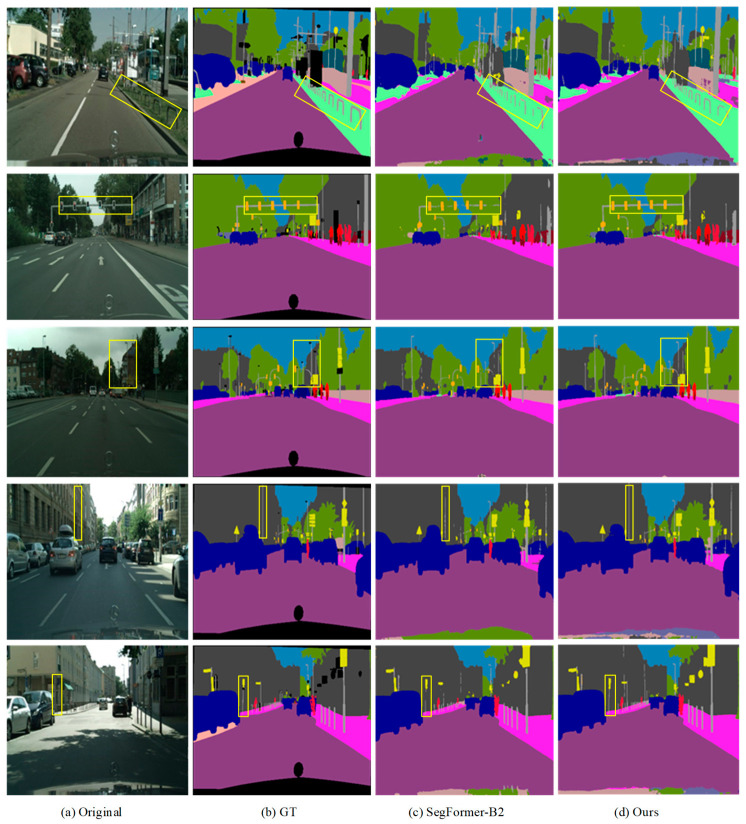
Segmentation result visualization on the Cityscapes dataset. Yellow boxes highlight representative traffic-scene objects or regions for visual comparison.

To visually validate the effectiveness of the proposed method, qualitative comparisons are conducted between the improved method and the original SegFormer-B2 on the Cityscapes dataset. The results are illustrated in Figure 5, where the highlighted regions are further analyzed below. As shown in the first row of Figure 5, SegFormer-B2 fails to effectively segment the fence within the greenbelt region. This is mainly because such objects have weak feature representations and are easily overwhelmed by complex background information, thereby increasing the segmentation difficulty. Furthermore, as shown in the second, third, fourth, and fifth rows of Figure 5, the baseline model also struggles to accurately segment small-scale objects such as poles and slender traffic signs. In contrast, the proposed method is able to accurately identify and segment these thin and elongated objects, as shown in Figure 5. Moreover, the predicted boundaries are smoother and more continuous, which further demonstrates the effectiveness and superiority of the proposed method in fine-grained object segmentation tasks.

#### 4.4.2. Experimental Results on the CamVid Dataset

To validate the performance of the proposed method on the CamVid dataset, further quantitative comparisons with several representative methods were conducted, and the results are reported in Table 6. Owing to the relatively smaller image resolution of the CamVid dataset, the proposed method achieves favorable segmentation performance, with a PA of 95.06%, an mPA of 91.33%, and an mIoU of 82.97%. Compared with methods such as DeepLab-V3+, PIDNet-Small, and SegFormer-B2, the proposed method shows certain advantages in overall segmentation performance. In particular, compared with the direct baseline SegFormer-B2, the mIoU is improved from 80.65% to 82.97%, yielding a gain of 2.32 percentage points, which indicates that the proposed method can effectively improve the segmentation quality of fine-grained targets and boundary regions within the current framework. In addition, compared with larger models such as DeepLab-V3+ and HyperSeg-L [31], the proposed method achieves higher segmentation accuracy with a relatively lower number of parameters, demonstrating a certain overall advantage in balancing accuracy and model complexity.

In addition, to evaluate the performance differences across categories—particularly the capability of modeling thin structures such as poles, traffic signs, and road boundaries—a per-class mIoU comparison is conducted between the proposed method and the baseline SegFormer-B2 on the CamVid dataset. The corresponding results are presented in Table 7.

To further investigate the category-level performance of the proposed method on the CamVid dataset, particularly its capability in modeling thin-structure objects, a per-class mIoU comparison is conducted between the proposed method and the baseline SegFormer-B2. As shown in Table 7, the proposed method achieves improvements across all categories, with the overall mIoU increasing from 80.65% to 82.97%. Notably, for typical fine-grained categories such as pole, traffic sign (signsymbol), pedestrian, and bicyclist, the mIoU gains range from approximately 3% to 5%. In contrast, for large-area categories such as sky, building, road, pavement, and car, the performance improvements are relatively modest, generally within about 1%. These results indicate that, on the CamVid dataset, the performance gains of the proposed method are also primarily concentrated on thin structures and boundary-sensitive categories, while maintaining moderate improvements for large background or dominant regions. This trend is consistent with the observations on the Cityscapes dataset, further demonstrating the generalization capability of the proposed method for fine-grained structure segmentation across different datasets.

**Figure 6:**
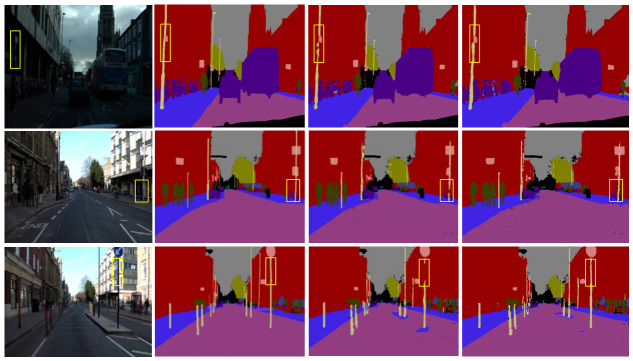

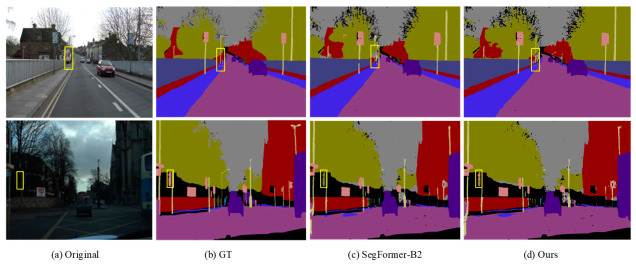
Segmentation result visualization on the Camvid dataset. Yellow boxes highlight representative traffic-scene objects or regions for visual comparison.

Based on the above per-class mIoU analysis, qualitative comparisons are further conducted on the CamVid dataset to visually evaluate the segmentation performance of the proposed method on thin and elongated objects. Specifically, the proposed method is compared with SegFormer-B2, and the results are illustrated in Figure 6, where the highlighted regions are further analyzed below. As shown in the first, second, and third rows of Figure 6, for thin and elongated targets such as traffic lights and lamp posts, SegFormer-B2 produces discontinuous segmentation results with fragmented boundaries. In contrast, the proposed method better preserves the structural continuity of these objects, yielding smoother and more complete segmentation outputs. In the fourth row of Figure 6, the baseline model incorrectly merges a narrow sidewalk region at the road–pavement boundary (highlighted by the yellow box) with adjacent large homogeneous regions, resulting in blurred semantic boundaries. By contrast, the proposed method effectively suppresses such semantic adhesion and restores the correct structural separation and clear boundaries. Furthermore, in the fifth row of Figure 6, the baseline SegFormer-B2 misclassifies a traffic light as background, whereas the proposed method demonstrates stronger semantic discrimination capability and is able to correctly segment the traffic light. In summary, the proposed method exhibits superior accuracy and robustness in segmenting small-scale and thin elongated objects in complex traffic scenes.

## 5. Discussion and Conclusions

### 5.1. Discussion and Limitations

To address the challenges of detail loss and insufficient contextual modeling for thin and small objects in traffic-scene semantic segmentation, a discrepancy-aware boundary-enhanced semantic segmentation method is proposed. By incorporating the Gated Collaborative Context Module (GCCM), the Frequency–Edge Guided Enhancement Module (FEGE), and the Discrepancy-aware Pixel-Adaptive Gating Fusion module (D-PagFM), the proposed framework effectively exploits multi-scale hierarchical features, thereby improving the representation of fine-grained targets and thin structures. Qualitative and quantitative results on the Cityscapes and CamVid datasets demonstrate that the proposed method improves the segmentation performance of small and thin objects while producing smoother and more precise object boundaries.

Compared with existing methods, the proposed framework provides a more effective balance between local detail preservation and global semantic modeling. In particular, GCCM enhances semantic completeness for fine-grained targets, FEGE improves the recovery of boundary and high-frequency structural information, and D-PagFM strengthens the robustness of cross-scale feature fusion by suppressing the interference of locally inconsistent features. The improvements are especially evident in boundary-sensitive and fine-grained categories, indicating that the proposed method is effective for handling structurally complex urban traffic scenes.

### 5.2. Limitations and Future Work

Despite the encouraging results, the proposed method still has several limitations. First, the introduction of multiple enhancement and fusion modules inevitably increases model complexity, which may limit its applicability in real-time or resource-constrained deployment scenarios. Second, although the proposed framework achieves consistent improvements on the Cityscapes and CamVid datasets, its generalization ability under more challenging conditions, such as adverse weather, nighttime scenes, and large domain shifts, has not yet been systematically validated. Third, while the proposed fusion strategy is beneficial for fine-grained and boundary-sensitive categories, its effectiveness may still be constrained in cases involving extremely small objects, severe occlusion, or highly ambiguous structures. In addition, the discrepancy-aware fusion mechanism adopted in this work serves as a practical proxy for unreliable feature interactions rather than a principled probabilistic uncertainty estimation framework. Therefore, further efforts are still needed to improve both the theoretical rigor and the applicability of the proposed method in more complex traffic-scene environments.

Future work will focus on three directions. First, the efficiency of the proposed framework will be further improved to better support real-time traffic-scene perception. Second, the current method will be extended to semi-supervised settings in order to reduce the dependence on large-scale pixel-level annotations. Third, multi-modal information, such as RGB–thermal and RGB–depth data, will be incorporated to enhance robustness under challenging environmental conditions. In addition, more principled reliability-aware modeling strategies will be explored to further improve cross-scale feature fusion and strengthen the generalization capability of the framework.

## Figures and Tables

**Table 1:** Ablation experiments on the Cityscapes dataset.

Baseline	GCCM	FEGE	D-PagFM	Params (M)	PA (%)	mPA (%)	mIoU (%)
√	−	−	−	27.36	96.06	86.15	77.91
√	−	√	−	35.21	96.22	86.43	78.96
√	√	−	−	31.12	96.23	87.15	79.0
√	−	−	√	29.14	96.2	87.18	79.5
√	√	√	−	38.97	96.3	87.01	79.42
√	−	√	√	37.00	96.33	87.5	79.68
√	√	−	√	32.91	96.33	86.79	79.35
√	√	√	√	40.76	96.37	87.95	80.08

Note: √ indicates that the corresponding component is used, whereas − indicates that the corresponding component is not used.

**Table 2:** Internal ablation experiments of the D-PagFM module.

Baseline	PagFM	D-PagFM	Params (M)	PA (%)	mPA (%)	mIoU (%)
√	−	−	27.36	96.06	86.15	77.91
√	√	−	28.70	96.24	87.05	79.35
√	−	√	29.14	96.2	87.18	79.5

Note: √ indicates that the corresponding component is used, whereas − indicates that the corresponding component is not used.

**Table 3:** Ablation Results of the Similarity–Discrepancy Joint Gating Mechanism.

Model	Params (M)	PA (%)	mPA (%)	mIoU (%)
Sim-PagFM	29.14	96.28	86.86	79.27
Conf-PagFM	29.14	96.26	86.52	79.13
Joint-PagFM	29.14	96.26	87.04	79.41
D-PagFM	29.14	96.2	87.18	79.50

**Table 4:** Performance comparison of different algorithms on the Cityscapes dataset.

Model	Params (M)	PA (%)	mPA (%)	mIoU (%)
DeepLab-V3+ [26]	58.75	95.67	83.49	75.04
BASeg [27]	63.99	95.76	86.64	80.39
Mask2Former-R50 [28]	62.1	-	-	79.4
Mask2Former-Swin-B [28]	66.1	-	-	83.3
SegFormer-B2 [30]	27.36	96.06	86.15	77.91
SegFormer-B3 [30]	47.3	96.22	87.51	81.7
STDC2-Seg100 [12]	12.95	95.75	84.53	76.93
SegNeXt-T [29]	4.3	-	-	79.8
SegNeXt-S [29]	13.9	-	-	81.3
PIDNet-Small [15]	7.6	96.11	86.11	78.60
Ours	40.76	96.37	87.95	80.08

**Table 5:** Per-Class mIoU Comparison Before and After Improvement on the Cityscapes Dataset.

Algorithm/Category	SegFormer-B2	Ours
road	98.23	98.3
sidewalk	85.38	85.59
building	92.47	93.14
wall	62.32	63.21
fence	59.94	61.91
pole	63.9	69.02
traffic light	70.53	73.79
traffic sign	78.48	81.74
vegetation	92.21	92.75
terrain	63.56	61.6
sky	94.94	95.2
person	82.29	83.68
rider	61.54	64.57
car	94.95	95.36
truck	82.32	84.44
bus	82.63	89.1
train	71.17	80.51
motorcycle	67.6	69.26
bicycle	76.22	78.32
mIoU	77.93	80.08

**Table 6:** Performance comparison of different algorithms on the Camvid dataset.

Model	Params (M)	PA (%)	mPA (%)	mIoU (%)
DeepLab-V3+ [26]	58.75	94.22	85.16	78.03
HyperSeg-L [31]	67.95	94.36	85.72	77.95
PIDNet-Small [15]	7.6	95.26	86.64	80.01
Segformer-B2 [30]	27.36	94.22	90.85	80.65
Ours	40.76	95.06	91.33	82.97

**Table 7:** Per-Class mIoU Comparison Before and After Improvement on the Camvid Dataset.

Algorithm/Category	Segformer-B2	Ours
sky	85.65	88.19
building	89.75	91.17
pole	53.35	58.37
road	97.6	97.69
pavement	89.15	89.26
tree	82.5	83.94
signsymbol	72.42	76.93
fence	76.62	79.79
car	90.0	91.82
pedestrain	69.65	73.01
bicyclist	80.44	82.54
mIoU	80.65	82.97

## Data Availability

The datasets analyzed in this study are publicly available. The Cityscapes dataset is available at https://www.cityscapes-dataset.com/, and the CamVid dataset is available at http://mi.eng.cam.ac.uk/research/projects/VideoRec/CamVid/, accessed on 6 April 2026. No new datasets were created in this study. Additional experimental results supporting the findings of this study are available from the corresponding author upon reasonable request.
